# Effect of the Bone Graft on the Surface Microhardness of Endodontic Biomaterials

**DOI:** 10.22037/iej.v13i2.14683

**Published:** 2018

**Authors:** Shahriar Shahi, Saeed Rahimi, Hamid Reza Yavari, Negin Ghasemi, Yashar Rezaie, Samira Mirzapour

**Affiliations:** a *Dental and Periodontal Research Center, Dental School, Department of Endodontics, Tabriz University of Medical Sciences, Tabriz, Iran;*; b *Department of Operative Dentistry, Dental School, Tabriz University of Medical Sciences, Tabriz, Iran; *; c *Dental School, Tabriz University of Medical Sciences, Tabriz, Iran*

**Keywords:** one Graft, Calcium-Enriched Mixture, Hardness, Mineral Trioxide Aggregate

## Abstract

**Introduction:**

During periapical surgery, using of bone products in large endodontic lesions, is a treatment option that could affect the properties of the retro-filling endodontic material. The aim of present study was to evaluate the effect of Osteon II bone powder on the surface microhardness of calcium-enriched mixture (CEM) and mineral trioxide aggregate (MTA).

**Methods and Materials:**

Each material was mixed and carried into 40 sterile custom-made plastic cylinders. Half of the samples in each group were exposed to Osteon II. All cylinders were submerged in simulated tissue fluid and incubated at 37^°^C and 100% relative humidity for 7 days. Surface microhardness values of each study group was attained using Vickers microhardness test. The data were analyzed statistically using two-way ANOVA and independent* t*-test at a significance level of 0.05.

**Results:**

The highest and lowest microhardness values were recorded in the MTA/without Osteon and MTA/with Osteon groups, respectively. Irrespective of the presence or absence of bone powder, the overall microhardness of CEM cement and MTA was not significantly different. In the MTA group, the presence of the powder resulted in a significant decrease (*P*<0.05) of the microhardness; however, its effect on CEM cement was not significant (*P*>0.05).

**Conclusion:**

Under the limitations of the present *in vitro* study, the presence of Osteon bone powder had no negative effect on the microhardness of CEM cement, contrary to its effect on MTA.

## Introduction

Apicoectomy is one of the treatment modalities for cases in which the orthograde root canal treatment has failed or is not possible. One of the factors affecting the success of surgical treatment is the placement of an appropriate retrofilling material in the root end cavity [1, 2]. This material should have proper sealing ability, be biocompatible, set in the presence of blood and moisture, and should not interfere with the regeneration of periapical tissues [3]. 

Mineral trioxide aggregate (MTA) is a calcium silicate-based biomaterial, which exhibits the majority of properties necessary for the use as a retrofilling material. Despite all the favorable properties reported for MTA, its setting time is long and its handling is difficult [4, 5]. Calcium-enriched mixture (CEM) cement is a biomaterial with different compositions of calcium and has applications similar to those of MTA. Its setting time is shorter than that of MTA and has better flowability than MTA; in addition, its manipulation is easier [6, 7]. The properties of CEM cement as a root-end filling material are comparable to those of MTA [8, 9]. 

Proper setting conditions should be provided for the setting of the retrofilling material to achieve clinical success [10]. In addition to the setting time, the acidic conditions of the surgical area due to the presence of inflammation and bleeding are considered one of the risk factors [11]. Another important factor that should be taken into account in this respect is the possible use of bone powders and their effects on the physical properties of the retrofilling material. Based on previous studies, large, through and through and apico-marginal lesions will heal better and faster when they are filled with a bone graft [12].

A study by Satto *et al.* [12] showed that presence of mineralized bone powder in the surgical area affect the setting and surface microhardness of MTA. Surface microhardness of a material is one of the characteristics directly related to the quality of the material’s setting. In fact, it depends on the hydration process and is affected by the pH and presence of ions in the environment [13]. Since one of the important consequences of improper setting is inadequate sealing, determination of surface microhardness of a retrofilling material in the presence of confounding factors such as bone powders is very important. The aim of this study was to determine and compare the surface microhardness of CEM cement and MTA in the presence of Osteon II bone powder.

## Materials and Methods

***Preparation of samples***

Eighty plastic cylinders, measuring 2 mm in internal diameter and 3 mm in height, were prepared and randomly assigned to 4 groups (*n*=20). In 40 samples, WMTA was used to fill the cylinders, and in the remaining 40, CEM cement was used to this end. The cylinders were placed on a glass slab and the materials were mixed according to manufacturers’ instructions. Then the materials were carried into the cylinders with an amalgam carrier and packed with a manual condenser; the upper and lower surfaces of the materials were made flush with the cylinders. The surface of the cylinder adjacent to the slab was marked. Then the filled cylinders were incubated at 37^°^ C and 100% relative humidity for one h to allow for initial hydration of the biomaterial. 

In the next stage, 4 plates with 20 wells were selected, and Osteen II bone powder, mixed with distilled water according to manufacturers’ instructions, was placed in two plates. As total of 1 mg of bone powder was placed in each well. After 10 min, 1 mL of synthetic tissue fluid (STF) was poured into each well. The cylinders were retrieved from the incubator and grouped as follows: 1) 20 cylinders of WMTA within the well containing bone powder and STF, 2) 20 cylinders of CEM cement within the well containing bone powder and STF, 3) 20 cylinders of WMTA within the well containing STF and 4) 20 cylinders of CEM cement within the well containing STF.

The samples were incubated at 37^° ^C and 100% relative humidity for two weeks, during which the plates were shaken gently every day for even distribution of ions in all the wells.

***Determination of microhardness***

The samples were retrieved from the plates for microhardness test and their surfaces were polished with silicone carbide-based sand (300, 600, 1200 and 2400 grits). Then the samples were rinsed in distilled water to eliminate debris resulting from the polishing procedure. Finally, the samples were dried with an air syringe. In order to carry out Vickers microhardness test, an indenter with a square base and pyramidal tip exerted a 300-g force for 10 sec on the surface of the samples so that microhardness in term of kgf/mm^2^ was displayed on the digital reading apparatus of the test equipment. The procedure was carried out on 3 points of each sample more than 1 mm apart from each other and from the cylinders margins. It should be pointed out that the force was applied to the surface of each cylinder that was not adjacent to the glass slab during packing of the material. The mean microhardness of these three points was recorded as the microhardness of each sample. 

***Statistical analysis***

The mean±standard deviation of microhardness of the study groups was calculated. Kolmogorov-Smirnov test was used to evaluate normal distribution of data. Since data was distributed normally and variances were equal, two-way ANOVA was used to evaluate the significance of the effect of material type (MTA or CEM cement) and the presence and absence of bone powder on microhardness. Independent *t-*test was used within each study group at a significance level of 0.05. 

## Results

The highest and lowest microhardness values were recorded in MTA/without Osteon and MTA/with Osteon groups, respectively. Table 1 presents the values recorded in each study group. The results of two-way ANOVA showed that the type of the material had no effect on microhardness (*P*=0.8); however, the presence or absence of bone powder had a significant effect on microhardness (*P*=0.03). Irrespective of the presence or absence of bone powder, the overall microhardness of CEM and MTA were no significantly different (*P*=0.7). In the MTA group, the presence or absence of bone powder exerted a significant effect on microhardness (*P*=0.001); however, its effect on CEM cement was not significant (*P*=0.8).

**Table 1:** Mean (SD) of the microhardness of study groups

**Material **	**Osteon**	**Mean (SD)**
**MTA**	no	33.28 (5.84)
	yes	26.03 (4.23)
**CEM**	no	32.50 (4.93)
	yes	31.24 (2.94)

It was shown that both independent variables of the type of retrofilling material and the presence of bone powder in the environment caused significant changes in surface microhardness at different levels (*P*<0.001). The interaction effect of these variables was significant (*P*=0.03).

## Discussion

The present study was undertaken to evaluate the effect of Osteon II mineralized bone powder on the surface microhardness of CEM cement and MTA. The results showed the negative effect of bone powder on the MTA surface microhardness; however, its effect on CEM cement surface microhardness was not significant. 

Use of bone powder in periapical surgery in cases such as large lesions has been suggested to achieve rapid healing of bony lesions. In such conditions, the retrofilling material used at the root-end cavity will come into direct contact with bone powder, which might affect the physical properties of the material [10]. 

In the present study, microhardness of two endodontic biomaterials was evaluated in the presence of bone powder. Microhardness indicates the strength of the material and in fact it shows its proper setting. Complete setting of the retrofilling material is a prerequisite for achieving a proper seal by the surgical retrofilling material. In this study Vickers test was used to evaluate microhardness, which is a commonly used and reliable test and has been used repeatedly in retrospective studies. 

The biomaterials used in this study consisted of CEM cement and MTA. Both these materials are calcium-based cements and the difference is the presence of phosphate in the chemical structure of CEM cement [14]. The physical and biologic properties of these two materials are similar and their efficacy has been shown in different areas of root canal treatment, such as their use as a retrofilling material [8, 9].

Various studies have evaluated MTA microhardness by taking into account different factors that are involved. Based on the results of these studies, an acidic environment, being exposed to FBS, the presence of blood and being mixed with propylene glycol and exposure to chlorhexidine and hypochlorite results in a decrease in its microhardness [13, 15-21]; however, no published article is available on the microhardness of CEM cement. One of the factors that can affect microhardness is the thickness of the retrofilling material, which was 3 mm in all the samples in the present study. The highest hardness has been reported for CEM cement with a thickness of 4 mm, with a decrease in hardness with an increase in the thickness of MTA and CEM cement [22]. 

In the present study, plastic cylinders were used instead of acrylic resin cylinders in the similar study [12] because the methyl methacrylate monomer released from the acrylic resin might affect the physicochemical properties of the retrofilling material. Since differences in the force used to pack the material might affect microhardness of the material, the materials were placed by one operator with the use of one condenser with a specific size. The plates were shaken gently every day in order to create a homogeneous ionic environment; in addition, STF was used to simulate the periapical tissue conditions because the interstitial fluids contact the retrofilling material in the real clinical conditions. 

The results of the present study showed the negative effect of the presence of bone powder in the area on MTA. MTA is hydrophilic cement and sets by hydration of powder particles. The hydration products of MTA are calcium silicate hydrate and calcium hydroxide [12]. The calcium ions released from the mineralized Osteon II grafts saturate the environment and prevent the release of the two chemical compositions above. In fact, they interfere with the hydration process of MTA. On the other hand, calcium hydroxide released due to the setting process of MTA synthesizes hydroxyapatite on the surface of MTA with the phosphate found in the environment. The presence of apatite deposits on the surface of MTA is another factor affecting microhardness and the wet condition improve the microhardness of MTA [23]. The mineralized graft has apatite crystals on its surface that serve as templates for the deposition of calcium and phosphate for the formation of hydroxyapatite; phosphate deposits are formed on the bone powder particles rather than on MTA [12]. 

Contrary to MTA, CEM cement was not negatively affected by bone powder, which might be attributed to the presence of phosphate in the chemical composition of CEM cement, which can form hydroxyapatite on its surface contrary to MTA; on the other hand, the setting time of CEM is shorter than that of MTA [24] and it is affected by the negative effects of bone powders at shorter durations. According to the results reported by Rahimi *et al.* [25], similar to CEM the microhardness of Biodentine was not affected with mineralized bone powder.

It should always be remembered that inflammation and the acidic environment resulting from it and also the presence of hemorrhage in that area are the confounding factors in the microhardness of the retrofilling material, which were not included in the present study. In addition, ionic exchange in the real surgical procedure was not simulated. Microhardness was evaluated in a one-week period. Previous studies have shown that the strength of MTA increases over time, which should be considered in future studies. 

Therefore, to reach a conclusion that can be extended to the clinic, further studies should be carried out at longer periods and by including all the factors affecting the physical properties of biomaterials.

## Conclusion

Under the limitations of the present study, when mineralized bone powder is used, CEM is a better choice than MTA.
